# A case of type B lactic acidosis as a complication of chronic myelomonocytic leukaemia: a case report and review of the literature

**DOI:** 10.1186/1752-1947-9-16

**Published:** 2015-01-14

**Authors:** Andrew John Gardner, John Griffiths

**Affiliations:** 13 Faculty of Medicine, Oxford University, John Radcliffe Hospital, Oxford, OX3 9DU UK; 14 Nuffield Department of Anaesthetics, John Radcliffe Hospital, Oxford, OX3 9DU UK; 15 Keble College, Oxford, OX1 3PG UK

**Keywords:** Acidosis, Chronic myelomonocytic leukaemia, Haematological malignancy, Type B lactic acidosis

## Abstract

**Introduction:**

Type B lactic acidosis represents a rare and often lethal complication of haematological malignancy. Here, we present a patient who developed a type B lactic acidosis presumably due to a concurrent chronic myelomonocytic leukaemia. Upon swift initiation of cytoreductive chemotherapy (doxorubicin), the lactic acidosis was rapidly brought under control. This case adds to the literature reporting other haematological malignancies that can cause a type B lactic acidosis and its successful treatment.

**Case presentation:**

We report the case of a 77-year-old Caucasian man brought to our Accident and Emergency department following an unwitnessed collapse; he was found surrounded by coffee-ground vomit. Although haemodynamically stable on admission, he rapidly deteriorated as his lactic acid rose. An initial arterial blood gas revealed a pH of 7.27 and lactate of 18mmol/L (peaking at 21mmol/L).

**Conclusions:**

A high degree of clinical suspicion for haematological malignancy should be held when presented with a patient with lactic acidosis in clinical practice, even without evidence of poor oxygenation or another cause. Treatment with emergency chemotherapy, in lieu of a definitive diagnosis, was rapidly successful at lowering lactate levels within 8 hours. This may suggest a causal and perhaps direct relationship between lactic acid production and the presence of leukemic cells. Veno-venous haemofiltration had no apparent effect on reducing the lactic acidosis and therefore its benefit is questioned in this setting, especially at the cost of delaying chemotherapy. In the face of a life-threatening lactic acidosis, pragmatic clinical judgement alone may justify the rapid initiation of chemotherapy.

## Introduction

Lactate is the metabolic product of anaerobic glycolytic metabolism [1]. The sole route for lactate removal is its oxidative conversion back into pyruvate. Under normal conditions lactate clearance occurs primarily by the liver (80 to 90%) and kidneys [2]. However, lactic acidosis (LA) arises from a disruption in the balance between the production and breakdown of lactate. The state of LA has been defined as a pH of ≤7.35 and a plasma lactate concentration of ≥5mEq/L (≥5mmol/L) [3].

LA was originally divided into two categories by Cohen and Woods: type A and type B [4]. They associated type A with clinical evidence of a state of poor tissue oxygenation (for example shock, hypoxaemia), with the acidosis driven by either the overproduction or underutilisation of lactate. In type B there is no clinical evidence of inadequate tissue oxygen delivery which is associated with sepsis, malignancy, diabetes, thiamine deficiency and liver disease [5]. It represents a rare and often lethal complication of haematological malignancy. Recent theories pertaining to its origin include a shift to glycolysis by malignant cells and an impairment of lactate conversion by renal and hepatic metabolism. Friedenberg *et al*. [6] reported that 67 cases of type B LA have been cited in the literature to date [6]. Using the same search terms (performing a MEDLINE and PubMed – National Library of Medicine, Bethesda, MD, USA – search with the key words “type B lactic acidosis” and “leukaemia” or “lymphoma”, all years), we were able to identify a further nine individual cases [1, 2, 7–12], including our own.

**Figure 1:**
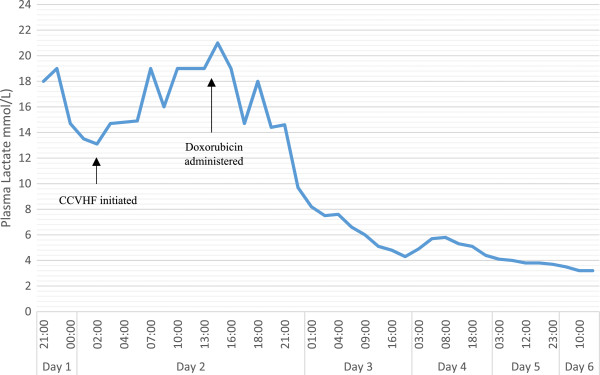
**Serum lactate levels were dramatically raised from admission until the initiation of doxorubicin.** Haemofiltration had no apparent effect on lactate levels. Lactate remained raised throughout admission, normal range is 0.4 to 1.7mmol/L, potentially due to a suspected subacute gastrointestinal bleed. CCVHF, continuous veno-venous haemofiltration.

Here, we present the rare case of a patient who developed a type B LA presumably due to concurrent chronic myelomonocytic leukaemia (CMML). No case to date has identified this association without the context of sepsis or transformation to acute myeloid leukaemia (AML) [6, 9]. Clinical uncertainty arose over the cause of the LA due to the paucity of literature on this association. This is likely to have contributed to a delay in the initiation of cytoreductive chemotherapy (doxorubicin) as a treatment option. When initiated, however, in lieu of a definitive diagnosis and without a benefit from haemofiltration, a single dose of doxorubicin was rapidly successful at lowering lactate levels within 8 hours (Figure 1).

## Case presentation

This is the case of a 77-year-old Caucasian man who presented to our Accident and Emergency department after he was discovered collapsed and surrounded by coffee-ground vomit (Table 1). Over the last 6 weeks, there had been a history of generalised malaise, anorexia, rapid weight loss, as well as epigastric pain and vomiting following meals (treated in general practice with ranitidine 150mg, once daily). There was no significant past medical history aside from long-standing, well-controlled, hypertension (atenolol 50mg and losartan 50mg, both once daily). No abnormalities were found on examination, including melena. A computed tomography scan (head) demonstrated a concomitant chronic subdural haematoma with midline shift of unknown cause. Initial differential diagnoses included a gastric malignancy, myelodysplasia and an upper gastrointestinal bleed.

**Table 1:** **Timeline**

Previous 6 weeks:	generalised malaise, anorexia, rapid weight loss, as well as epigastric pain and vomiting following meals
Day 1:	Admission to Accident and Emergency with collapse and coffee-ground vomit, transfer to Intensive Care Unit
Day 2 (02:00):	Continuous veno-venous haemofiltration instituted
Day 2 (14:00):	Doxorubicin (50mg, intravenous, once), rasburicase and Pabrinex® (vitamins thiamine, riboflavin, pyridoxine, ascorbic acid and nicotinamide) administered
Day 2 (15:00)	Plasma lactate peaks at 21.0mmol/L
Day 3 (20:00)	Plasma lactate plateaus at approximately 4mmol/L
Day 7:	Transferred to haematology
Day 15:	Surgical evacuation of haematoma
Day 16:	Transfer to Neurological Intensive Care Unit
Day 20:	Withdrawal of care in patient’s best interests
Day 21:	Patient dies, 02.17 a.m.

On admission, he was Glasgow Coma Scale (GCS) 14, temperature was 36.7°C, heart rate was 110 beats/minute, arterial blood pressure 152/50mmHg, with respiratory rate of 23 and a blood oxygen saturation of 99% self-ventilating on 21% oxygen. An initial arterial blood gas revealed a lactate of 18mmol/L, bicarbonate of 11mmol/L and a base excess of −19 (pH 7.27): a LA. His delta ratio was 1.9:1 consistent with a pure LA in which the delta ratio averages 1.6:1. This result and a rapid physiological decline prompted admission to our Intensive Care Unit (ICU).

His initial blood film demonstrated features of myelodysplastic syndrome (dysplasia, thrombocytopenia, occasional blast cells); white cell count 25.48×10^9^/L, neutrophils 9.98, lymphocytes 2.4, and monocytes 7.58. Antibiotics were given as per the local protocol for neutropenic sepsis: Tazocin® (piperacillin-tazobactam) and gentamicin. While sepsis was not entirely discounted, blood cultures were negative and a chest radiograph revealed no abnormalities. After 6 hours of ICU admission, continuous veno-venous haemofiltration was instituted for the first 60 hours of admission. A bone marrow aspirate was compatible with CMML but suggested no acute transformation to AML. Despite initial reservations to ascribe the cause of the LA to the haematological malignancy, emergency chemotherapy (doxorubicin, 50mg, intravenous, once) was instituted, alongside rasburicase and empiric intravenous Pabrinex® (vitamins thiamine, riboflavin, pyridoxine, ascorbic acid and nicotinamide) therapy. His lactic acid peaked at 21mmol/L (Figure 1) 1 hour after (15:00 hours) and had plateaued at a nadir of approximately 4mmol/L by 20:00 hours the next day (Figure 1). Thiamine levels were retrospectively reported as 458nmol/L. Initial results suggested no liver or renal dysfunction: creatinine (123mmol/L) and urea (22.5mmol/L), alanine transaminase (20U/L), aspartate transaminase (91U/L) and albumin (47g/L).

On day seven, he was transferred to haematology awaiting leukocyte and platelet counts to recover. After 11 days, the decision was made to surgically evacuate his subdural haematoma to give the best chance for a neurological recovery (GCS fluctuated, with a baseline of nine). Post-surgery, he showed minimal improvement in our Neurological ICU. The decision was made, with the family, to withdraw care on day 21.

## Discussion

Despite the severity of type B LA, little is known about its pathophysiological basis and there is no consensus on empirical treatment [2]. Review of the case literature reveals it to be a well-recognised phenomenon, despite its rarity, highlighting the need for development of clinical practice aimed at the rapid diagnosis and treatment of this condition.

### Mechanisms

Lactate is converted to and from pyruvate in the liver and kidney. In acidosis, the renal clearance of lactate predominates as hepatic uptake is significantly impaired. At pH 7.45 the kidneys clear 16% of lactate, whereas at pH 6.75 this rises to approximately 44%; compensating for half of the reduced hepatic function [13, 14]. Hence, metabolic impairment of these organs either by ischaemic damage or neoplastic infiltration could precipitate an impediment of lactate clearance [5]. Despite inconsistent laboratory or radiographic findings, metabolic disruption is a common occurrence among patients with haematological malignancies and those on critical care wards. However, type B LA and liver or renal failure do not always coexist (neither were apparent in our case), therefore another mechanism must also contribute to LA.

The Warburg effect is the observation that tumour cells favour glycolysis instead of oxidative metabolism, even at normal oxygen concentrations (‘aerobic glycolysis’) [15]. This fact underlies the near 90% sensitivity and specificity of fluorodeoxyglucose positron emission tomography [16]. Although a relatively inefficient metabolic process, it is hypothesised the glycolytic phenotype initially arises as an adaption to local hypoxia [16]. Likewise, hypoxic pressure may also arise due to the impaired perfusion of healthy tissues either by solid tumour infiltrate or leukemic micro-emboli [6].

Hexokinase and insulin-like growth factors (IGFs) are regularly overexpressed in malignant cells and promote glycolysis [3]. Hexokinase catalyses the initial rate-limiting step in glycolysis and when present at high concentrations can promote glycolytic metabolism, even in the presence of oxygen. In addition, IGFs can mimic insulin, an activator of hexokinase. Similarly, the role of the hypoxia-inducible factor genes, which allow cellular adaption to hypoxia can upregulate glycolysis and are known to drive neoplastic growth in some tumour types (for example Von Hippel–Lindau disease) [5]. Nevertheless, the promotion of aerobic glycolysis in haematological malignancies may precipitate uncontrollable lactate production.

A shift from oxidative to glycolytic metabolism may also be mediated by inflammatory cytokines released by, and in response to, haematological malignancy. For example, tumour necrosis factor alpha (TNFα) is often correlated to plasma lactate concentration and have been shown to inhibit pyruvate dehydrogenase (PDH) [3]. However, the causal relationship between TNFα and LA has not been elucidated; for example, *in vitro* hyperlacticacidemia itself has been shown to promote the transcription of TNFα [17]. PDH is an important enzyme in the production of acetyl-CoA, which enters the Krebs cycle and ultimately drives the electron transport chain as part of oxidative respiration. Disruption of this process may drive lactate production and, ultimately, LA [6].

Another described mechanism postulates deficiencies in thiamine may drive LA. Thiamine is required for PDH enzymatic activity [18]. One case reported reversal of LA with thiamine supplementation to a patient receiving total parenteral nutrition [19].

In sepsis, with rapid leukocyte production, excessive pyruvate and lactate production occurs due to an accelerated glycolytic rate, independent of anaerobic glycolysis and impairment of PDH [20]. Lactate is produced by leukocytes, which have few mitochondria and little capacity to metabolise pyruvate aerobically [21]. Haematological malignancies could conceivably drive LA by similar mechanisms. Likewise, a high rate of cellular apoptosis may also drive compensatory glycolysis due to a loss of mitochondrial function; for example, this occurs in tumour lysis syndrome [6, 22].

### Therapy prospects

On the premise that neoplastic cells are driving the type B LA, cytoreductive chemotherapy is the most widely used therapeutic strategy. Our report and other clinical data in the literature are supportive of this, as resolution of LA often occurs with treatment and reoccurs with a relapse of malignancy [23–26]. While this occurred in this case within 7 hours of doxorubicin administration, causality cannot be definitely determined. A repeat bone marrow aspiration or blood film was not performed, with reduction of leukemic status only being inferred from the full blood count.

The benefit of empiric Pabrinex® (vitamins thiamine, riboflavin, pyridoxine, ascorbic acid and nicotinamide) therapy is unknown, although the patient was not deficient in thiamine on testing. Haemodialysis aimed at removing excess lactate has shown some efficacy as an adjunct to chemotherapy, although its efficacy remains controversial [27]. No liver or renal dysfunction was apparent in this case. Despite haemodialysis, the lactate levels of our patient remained stubbornly high, peaking at 21mmol/L. The persistent elevation of his lactate was possibly due to a suspected concurrent gastrointestinal bleed. Therefore, the contributory effect of haemodialysis in this case, as well as treating type B LA in general, may be questioned.

## Conclusions

Type B LA is a life-threatening complication of haematological malignancies associated with a poor prognosis. A high degree of clinical suspicion for haematological malignancy should be held when presented with a patient with LA, especially without evidence of poor oxygenation or another cause. This report adds CMML to other haematological malignancies which are recognised direct causes of type B LA. The initiation of chemotherapy targeting the underlying malignancy rapidly reversed the LA. This may suggest a causal and perhaps direct relationship between lactic acid production and the presence of leukemic cells. In the face of a life-threatening LA, pragmatic clinical judgement alone may justify the early initiation of chemotherapy.

## Consent

Written informed consent was obtained from the patient’s next-of-kin for publication of this case report and any accompanying images. A copy of the written consent is available for review by the Editor-in-Chief of this journal.

## Abbreviations

AML: Acute myeloid leukaemia
CMML: Chronic myelomonocytic leukaemia
GCS: Glasgow Coma Scale
ICU: Intensive Care Unit
IGF: Insulin-like growth factor
LA: Lactic acidosis
PDH: Pyruvate dehydrogenase
TNFα: Tumour necrosis factor alpha.
